# Prenatal exposure to persistent organic pollutants and child overweight/obesity at 5-year follow-up: a prospective cohort study

**DOI:** 10.1186/s12940-017-0338-x

**Published:** 2018-01-18

**Authors:** Hilde B. Lauritzen, Tricia L. Larose, Torbjørn Øien, Torkjel M. Sandanger, Jon Ø. Odland, Margot van de Bor, Geir W. Jacobsen

**Affiliations:** 1 Department of Public Health and Nursing, Faculty of Medicine and Health Sciences, Norwegian University of Science and Technology, Trondheim, Norway; 2 Department of Community Medicine, University of Tromsø – The Arctic University of Norway, Tromsø, Norway; 3 NILU-Norwegian Institute for Air Research, Fram High north research Centre for Climate and the Environment, Tromsø, Norway; 4 School of Health Systems and Public Health, University of Pretoria, Pretoria, South Africa; 5 Department of Environment and Health, VU University, Amsterdam, The Netherlands

**Keywords:** Perfluoroalkyl substances, Organochlorines, Childhood obesity, Non-monotonic dose-response relationship, Pregnancy, Endocrine disrupting chemicals, Skinfolds

## Abstract

**Background:**

Prenatal exposure to persistent organic pollutants (POPs), may influence offspring weight gain. More prospective epidemiological studies are needed to compliment the growing body of evidence from animal studies.

**Methods:**

Serum from 412 pregnant Norwegian and Swedish women participating in a Scandinavian prospective cohort study were collected in 1986–88, and analyses of two perfluoroalkyl substances (PFASs) and five organochlorines (OCs) were conducted. We used linear and logistic regression models with 95% confidence intervals (CIs) to evaluate the associations between maternal serum POP concentrations at 17–20 weeks of gestation and child overweight/obesity (body mass index (BMI) ≥ 85th percentile) at 5-year follow-up. Results were further stratified by country after testing for effect modification. We also assessed potential non-monotonic dose-response (NMDR) relationships.

**Results:**

In adjusted linear models, we observed increased BMI-for-age-and-sex z-score (β = 0.18, 95% CI: 0.01–0.35), and increased triceps skinfold z-score (β = 0.15, 95% CI: 0.02–0.27) in children at 5-year follow-up per ln-unit increase in maternal serum perfluorooctane sulfonate (PFOS) concentrations. We observed increased odds for child overweight/obesity (BMI ≥ 85th percentile) for each ln-unit increase in maternal serum PFOS levels (adjusted OR: 2.04, 95% CI: 1.11–3.74), with stronger odds among Norwegian children (OR: 2.96, 95% CI: 1.42–6.15). We found similar associations between maternal serum perfluorooctanoate (PFOA) concentrations and child overweight/obesity. We found indications of NMDR relationships between PFOS and polychlorinated biphenyl (PCB) 153 and child overweight/obesity among Swedish children.

**Conclusion:**

We found positive associations between maternal serum PFAS concentrations and child overweight/obesity at 5-year follow-up, particularly among Norwegian participants. We observed some evidence for NMDR relationships among Swedish participants.

**Electronic supplementary material:**

The online version of this article (10.1186/s12940-017-0338-x) contains supplementary material, which is available to authorized users.

## Background

The prevalence of childhood overweight and obesity (BMI ≥ 85th percentile) has increased dramatically over the past decades [1]. From 1990 to 2010, the global estimated prevalence of overweight and obesity among preschool children increased from 4.2% to 6.7% [1]. This trend is expected to continue, and the World Health Organization (WHO) predicts that 60 million preschool children worldwide (9.1%) will be overweight or obese by 2020 [1]. Childhood obesity is a risk factor for several chronic diseases later in life including diabetes, cardiovascular disease, musculoskeletal disorders, and some forms of cancer [2]. Dietary influences, a sedentary lifestyle, as well as possible gene-environment interactions are important determinants of the increasing obesity trends, but they do not completely account for the obesity epidemic [3]. An increasing body of evidence suggests that in utero exposure to endocrine disrupting chemicals (EDCs) may contribute to obesity development in children and adults [3, 4]. Animal and in vitro studies suggest that EDCs may cause obesity through interference with lipid metabolism to promote fat storage, by altering the metabolic set points, or modifying hormonal control of appetite and satiety [4]. Obesity may be programmed in the intrauterine period, and fetal exposure to certain EDCs may modify the epigenome of stem cells to preferentially produce more adipocytes at the cost of bone [5].

Several persistent organic pollutants (POPs), including perfluoroalkyl substances (PFASs) and organochlorines (OCs), are classified as EDCs [4]. PFASs and OCs are ubiquitous, persistent and bio-accumulative chemicals that have been detected in maternal serum throughout pregnancy and in cord blood at delivery. Although the use of some POPs is presently banned or restricted in many countries [6], adverse health outcomes related to background levels of POP exposures are still a major public health concern [7].

Compared to animal studies, prospective epidemiological studies investigating the association between maternal serum POP concentrations during pregnancy and offspring postnatal obesity are less extensive [8, 9]. For PFAS exposures, longitudinal studies have reported both positive [10–15] and no associations [16, 17]. For OCs, prenatal exposure to *p,p’*-dichlorodiphenyldichloroethane (*p,p’*-DDE) has been associated with increased body mass index (BMI) in infancy and childhood [8, 9], but less consistent findings are reported for associations with prenatal polychlorinated biphenyl (PCB) and hexachlorobenzene (HCB) [8, 9]. Most previous studies used anthropometric indices, such as BMI, as proxies for offspring body composition [18]. However, children with the same amount of body fat can have quite different BMI values. For this reason, skinfold thickness may be a more informative measure of body fat mass in children [19].

The current study includes 412 mother-child pairs from a Scandinavian prospective cohort study with participants from Norway and Sweden. We aimed to evaluate the associations between maternal serum POP concentrations in early pregnancy and offspring anthropometry, including child overweight/obesity at 5-year follow-up.

## Methods

### Study participants

**Fig. 1:**
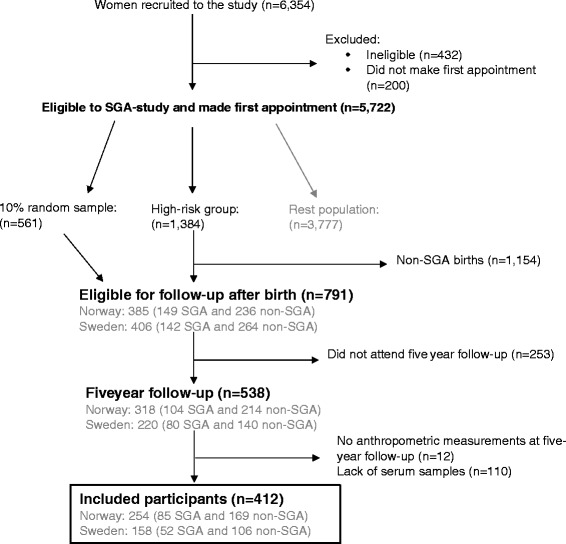
Flow chart of participants

This current study uses data from the U.S. National Institute of Child Health and Human Development (NICHD) Scandinavian Successive Small-for-Gestational Age births study (The SGA Study) [20]. The SGA Study is a large multi-center prospective cohort study conducted in Trondheim and Bergen (Norway) and Uppsala (Sweden) from 1986 to 1988. The SGA Study was designed to study longitudinal fetal growth, as well as perinatal and postnatal outcomes among mother and child [20]. In brief, all pregnant women (< 20 weeks gestation) in the study catchment areas who were expecting their 2nd or 3rd child were eligible for study inclusion and made the first appointment (*n* = 5722) (Fig. 1). Women with elevated risk for an SGA birth were intentionally oversampled. Risk factors for SGA birth included a previous low birth weight child, previous perinatal death, low maternal pre-pregnancy weight (< 50 kg), maternal smoking at conception and/or chronic maternal hypertension or renal disease. All high-risk pregnancies resulting in an SGA birth (birth weight below 10th percentile adjusted for sex and parity), and a 10% random sample of the study population were invited for follow-up when children were five years of age (*n* = 791). Of these, 534 (68%) attended the 5-year evaluation. In the current study, 412 mother-child pairs (137 SGA births and 275 non-SGA births) were included in the analyses (Fig.1).

### Exposure assessment of maternal serum POP concentrations

According to study protocol (1986–88), maternal serum samples were collected in the 2nd trimester (gestational week 17–20) and stored at minus 80 °C for later analysis. Analyses of maternal serum PFAS and OC concentrations were performed.

#### PFAS analyses

The PFAS analyses were performed at the laboratories of Norwegian Institute for Air Research, Tromsø, Norway (NILU). Maternal serum samples were quantified for two target analytes including perfluorooctanoate (PFOA) and perfluorooctane sulfonate (PFOS). Detailed information about the sample preparation, extraction method, analytical method, reagents and instrumentation is previously reported [21, 22]. Maternal serum PFAS concentrations were determined using sonication-facilitated liquid-liquid extraction, activated ENVI-carb clean-up [23], and analyzed by ultrahigh pressure liquid chromatography triple-quadruple mass-spectrometry (UHPLC-MS/MS). Participation in the AMAP Ring Test [24] ensures that the uncertainties of the analysis are within ±15–20% of the assigned values.

#### OC analyses

Maternal serum OC concentrations were analysed at the Institut National de Santé Publique du Quebec, Centre Toxicologie, Quebec. Several OCs were measured, including hexachlorobenzene (HCB), oxychlordane, polychlorinated biphenyl (PCB) 52, 101, 118, 153, 156, 170, and 180, *p,p’*-dichlorodiphenyldichloroehylene (*p,p’*-DDE), *p,p’-*dichlorophenyltrichloroethane (*p,p’*-DDT), *β*-hexachlorohexane (*β*-HCH) and *trans*-nonachlor (*t-*NC). In short, 0.5–1 ml serum sample was extracted using hexane (2 × 6 ml), ethanol (2 ml) and saturated ammonium sulphate solution (2 ml). This method is a slight modification of the one described by Sandanger et al. [25], where the samples were cleaned up using 1 g of activated fluorisil on an automated Liquid handler system before GC-MS analysis [26]. Uncertainties of the analyses are within ±15–20% of the assigned values, which are confirmed by participation in the AMAP Ring Test [24]. Lipids were enzymatically determined and the summed lipid amounts were calculated based on triglycerides and cholesterol measurements using the following formula:

Total lipids = 1.33*triglycerides +1.12*cholesterol +1.48 (g/l) [27]. This formula showed good correlation with complete formulas including phospholipids [28].

We report PCB 153 as a proxy for total PCBs, and excluded *p,p*-DDT because of low limit of detection (LOD) (> 50% of samples < LOD). LODs are listed in Table 2. Values below LOD were replaced by LOD/√2.

### Outcome assessment of child overweight/obesity

Child weight was recorded at 5-year follow-up in the clinic by trained professionals using a standard procedure. Standing weight was recorded to the nearest 100 g. Standing height was measured according to standard procedures and recorded to the nearest 0.1 cm [29]. BMI was calculated from weight in kilograms (kg) divided by height in meters squared (kg/m^2^). We used offspring age (in months), offspring sex and offspring BMI to calculate age-and-sex-specific BMI z-scores. BMI percentiles were based on the 2006 WHO child growth standards for children 5 years or younger [30], and the 2007 WHO growth standards for children and adolescents aged 5 to 19 years [18]. We assessed child age-and-sex-specific BMI z-scores as a continuous outcome at 5-year follow-up. We also analyzed child overweight/obesity at 5-year follow-up categorically (BMI ≥ 85th percentile for age and sex compared to BMI below the 85th percentile) [31]. Skinfold thickness was measured once using a Harpenden caliper (John Bull, British Indicators Ltd.) to the nearest 0.10 mm and 60 s after release of the grip to allow full tension placed on the compressed skinfold. Subscapular skinfold thickness was measured below the inferior angle of the left scapula, and triceps skinfold thickness was measured over the triceps in the middle of the left upper arm [29, 32]. We calculated age-and-sex-specific z-scores for triceps and subscapular skinfolds according to the Center for Disease Control and Prevention (CDC) 2000 Growth Charts for children from 1.5 to 20 years of age [33].

### Covariates

Information on maternal age, height, pre-pregnancy weight, education, smoking habits, previous breastfeeding duration and inter-pregnancy interval were collected via in-person interviews and self-report questionnaires during the original study period as per SGA Study protocol. Maternal pre-pregnancy BMI was calculated based on self-reported height and weight at first study visit. We calculated maternal weight gain up to 17 weeks of gestation as the difference between self-reported pre-pregnancy weight and clinically recorded weight closest to gestational week 17 (done by the woman’s own midwife or GP). Based on a known J-shaped association with adverse perinatal outcomes including restricted fetal growth [34], we categorized the inter-pregnancy interval as <18 months, 19–60 months and > 60 months since their last birth.

### Statistical analyses

Maternal serum PFAS and OC concentrations were logarithmically (ln) transformed to obtain normal distribution. We used wet weight maternal serum PFAS concentrations, and lipid-adjusted maternal serum OC concentrations [27].

We used multivariable linear regression with 95% confidence intervals (CIs) to examine the association between ln-transformed maternal serum concentrations of seven separate POPs (*PFASs:* PFOA, PFOS; *OCs:* PCB153, *p,p’*-DDE, HCB, *t*-NC and *β-*HCH) and offspring i) sex-and-age-specific z-scores for BMI at 5-year follow-up, and ii) sex-and-age-specific triceps and subscapular skinfolds at 5-year follow-up. We used multivariable logistic regression to estimate adjusted odds ratios (ORs) and 95% CIs for the association between maternal serum POP concentrations and child overweight/obesity (BMI z-scores ≥ 85th percentile for age and sex) at 5-year follow-up. We constructed a directed acyclic graph (DAG) to assess and select potential confounders (Additional file 1: Fig. S1). Prenatal growth was considered a mediator in the pathway between exposure to POPs and childhood overweight, due to positive associations between increasing prenatal levels and POPs and SGA birth in our study sample [35]. As adjustment for a mediator may introduce collider bias if there are shared unmeasured causes of both the mediator (SGA status) and the outcome (childhood overweight) [36], we did not include prenatal growth or SGA status in the multivariate analyses. The following variables were included in multivariable analyses as potential confounders: maternal age (continuous; years), maternal pre-pregnancy body mass index (BMI) (continuous: kg/m^2^), maternal education (categorical: < 9 years, 10–12 years, or ≥ 13 years), maternal smoking status at conception (categorical: 0, 1–9 or ≥ 10 cigarettes per day), previous breastfeeding duration (continuous: months), inter-pregnancy interval between the last two children (categorical: ≤ 18 months, 19–60 months, ≥ 61 months), and maternal weight gain from conception up to gestational week 17 (continuous: kilograms). The pooled analyses were further adjusted by country (Norway or Sweden). All models were tested for normality of residuals, heteroscedasticity, and multi-collinearity.

We examined linearity by scatter plots, assigning maternal serum POP concentrations to the horizontal axis, and measures of child adiposity to the vertical axis. Marginal relationships between maternal serum POP concentrations and offspring BMI z-scores at 5-year follow-up were assessed by non-linear regression using 3-knot restricted cubic splines and 95% CIs. We determined non-linear associations by examination of cubic spline graphs, and by the Wald test.

We had some missing data including 7.2% missing for both maternal weight gain up to gestational week 17 and previous breastfeeding duration. Among children, we had 7.0% missing data on subscapular skinfold thickness and 6.1% missing data on triceps skinfold thickness. Overall, 80% of participants had complete data on all variables. Missing data were assumed missing at random. We used chained multiple imputation [37, 38] to generate and compare five complete data sets. Complete case analyses widened the 95% CIs, but did not change the estimates substantially.

We evaluated possible effect modification by country and offspring sex based on a priori evidence from the literature [12, 35]. We conducted several sensitivity analyses to assess the robustness of our results. First, we did stratum-weighted analyses to ensure generalizability of our reported estimates to the contemporary pregnant population according to the prevalence of i) SGA births, ii) maternal pre-pregnancy overweight, and iii) maternal smoking at conception (See Additional file 1: Supplementary description S1 for details). Such weighted analyses are recommended for analyses with case-control data or in other way unbalanced populations that may be subject to selection bias [39]. Second, we additionally adjusted for maternal fish consumption during pregnancy among Norwegian participants (See Additional file 1: Supplementary description S2 for details). Finally, we considered a multi-pollutant model approach by mutually adjusting for maternal serum POPs that were found to be associated with offspring BMI.

All statistical analyses were conducted with SPSS statistical software, version 22 (IBM SPSS Inc. Chicago, IL) and Stata IC/13.1.

## Results

Overall, mean maternal age at study start was 29 years, with 69% of women expecting their second child, and 31% expecting their third (Table 1). Mean duration of previous breastfeeding was 7 months. On average, mothers gained 3.2 kg from conception up to gestational week 17. Overall, 10% of mothers were underweight (BMI < 18.5 kg/m^2^) at conception, and 9% were overweight or obese (BMI ≥ 25 kg/m^2^), with some variation between countries. A higher proportion of Norwegian mothers were underweight (BMI < 18.5 kg/m^2^) at conception compared to their Swedish peers (12% vs. 7%). A total of 53% of Norwegian mothers reported smoking at conception, compared to 33% of the Swedish mothers.

**Table 1:** Maternal and child characteristics by country of residence (*N* = 412)

Maternal characteristics	Total (*n* = 412)	Norway (*n* = 254)	Sweden (*n* = 158)	*p* ^a^
Maternal age (mean years (sd))	29.0(4.3)	28.8(4.3)	29.2(4.4)	0.364
missing n(%)	3(0.7)	1(0.4)	2(1.3)	
Maternal pre-pregnancy BMI (kg/m^2)^ n(%)				0.031
Underweight (BMI < 18.5)	42(10)	31(12)	11(7)	
Normal weight (BMI 18.5–25.0)	330(81)	205(81)	125(80)	
Overweight (BMI > 25.0)	38(9.3)	17(7)	21(13)	
missing n(%)	2(0.4)	1(0.4)	1(0.6)	
Maternal education (years) n(%)				0.011
less than 9	67(17)	36(14)	31(20)	
9–11	205(50)	142(56)	63(41)	
12 or more	135(33)	75(30)	60(39)	
missing n(%)	5(1.2)	1(0.4)	4(2.5)	
Maternal smoking at conception (number of cigarettes) n(%)				< 0.001
0	225(55)	120(47)	105(67)	
1–9	42(10)	35(14)	7(4)	
10 or more	145(35)	99(39)	46(29)	
Parity n(%)				0.245
1	285(69)	181(71)	104(66)	
2	127(31)	73(29)	54(34)	
Inter-pregnancy interval (years) n(%)				0.187
less than 1.5	102(25)	56(22)	46(29)	
1.5–5	225(55)	147(58)	78(49)	
5 or more	85(21)	51(20)	34(22)	
Previous breastfeeding duration (mean months (sd))	7.3(5.1)	7.5(5.2)	7.1(5.0)	0.546
missing n(%)	30(7.2)	13(5.1)	17(10.8)	
Maternal weight gain up to 17 weeks of gestation (mean kilograms (sd))	3.2(2.6)	3.1(2.6)	3.3(2.6)	0.601
missing n(%)	30(7.2)	28(11.0)	2(1.3)	
Child characteristics at birth				
Sex n (%)				0.987
Boys	211(51)	130(51)	81(51)	
Girls	201(49)	124(49)	77(49)	
Birth weight (mean grams (sd))	3445(592)	3401 (568)	3515 (625)	0.056
SGA status n(%)				0.908
non-SGA	275(67)	169(67)	106(67)	
SGA	137(33)	85(33)	52(33)	
Breastfeeding duration of the index child (mean months (sd))	6.5(3.6)	6.7(3.7)	6.0(3.2)	0.030
missing n(%)	16(3.9)	8(3.1)	8(5.0)	
Child characteristics at 5 year follow-up				
Exact age at five-year follow-up (mean years (sd)	5.2(0.2)	5.1(0.2)	5.4(0.1)	< 0.001
missing n(%)	1(0.2)	0(0)	1(0.6)	
Weight				0.708
Underweight (< 5th percentile)	12(3)	7(3)	5(3)	
Normal weight (5th–85th percentile.)	346(84)	211(83)	135(87)	
Overweight (> 85th percentile)	30(7)	21(8)	9(6)	
Obese (> 95th percentile)	22(5)	15(6)	7(4)	
missing n(%)	2(0.4)	0(0)	2(1.3)	
Subscapular skinfolds (mean mm (sd))	5.6(1.7)	6.0 (1.9)	5.1 (1.2)	< 0.001
Triceps skinfolds (mean mm (sd))	10.0(2.2)	10.4 (2.2)	9.2 (1.9)	< 0.001
Subscapular skinfold z-score	−0.01(0.8)	0.2(0.7)	−0.3(0.8)	< 0.001
missing n(%)	29(7.0)	16(6.3)	13(8.2)	
Triceps skinfold z-score	0.2(0.7)	0.3(0.6)	−0.1(0.7)	< 0.001
missing n(%)	25(6.1)	15(5.9)	10(6.3)	
BMI-for-sex-and-age z-score	0.1(1.0)	0.2(1.0)	0.1(0.9)	0.499
missing n(%)	2(0.4)	0(0)	2(1.3)	

*Sd* standard deviation, *BMI* body mass index, *SGA* small for gestational age

^a^*p*-values for comparison between the countries by using independent t-tests for continuous variables and χ^2^ tests for categorical variables

Children at 5-year follow-up were evenly distributed by sex (51% boys and 49% girls), wherein 1/3 were categorized as SGA births (reflecting the oversampling of SGA births for follow-up). Norwegian children had slightly lower birth weight (3401 vs. 3515 g), and were breastfed longer (6.8 vs. 6.0 months) than Swedish children. Norwegian children were also younger at 5-year follow-up (Norway: 61 months, Sweden: 65 months). A total of 55 children (12%) were considered overweight or obese at 5-year follow-up (Norway: 14%, Sweden: 10%). In our study population, Norwegian children had higher sex-and-age-adjusted z-scores of subscapular skinfold thickness (Norway: 0.18; Sweden: −0.32), and triceps skinfold thicknesses (Norway: 0.32; Sweden: −0.07) compared to Swedish children (Table 1).

Norwegian mothers had substantially lower median serum PFOA concentration (1.64 vs. 2.33 ng/ml), PFOS concentration (9.62 vs. 16.3 ng/ml), PCB 153 concentration (79.9 vs. 117 ng/g lipid) and *β*-HCH concentration (21.2 vs. 25.0 ng/g lipid) compared to Swedish mothers (Table 2). Norwegian mothers had higher median serum *t-*NC concentration (6.77 vs. 6.28 ng/g lipid) compared to Swedish mothers. Median maternal serum HCB concentrations (17.0 vs. 18.4 ng/g lipid) and *p,p’*-DDE concentrations (211 vs. 244 ng/g lipid) did not differ between countries (Table 2).

**Table 2:** Wet-weight levels of maternal serum PFASs, and wet-weight and lipid-adjusted levels of maternal serum OCs, by country (*n* = 412)

	Norway (*n* = 254)		Sweden (*n* = 158)		*p* ^a^	LOD	% > LOD
	Median	5th–95th perc.	Median	5th–95th perc.			
Wet-weight (ng/ml)							
PFOA	1.64	0.82–3.54	2.33	0.95–4.28	< 0.001	0.03	100
PFOS	9.62	3.78–24.6	16.3	7.17–30.5	< 0.001	0.03	100
PCB 153	0.46	0.26–0.83	0.68	0.37–1.10	< 0.001	0.01	100
*p,p’*-DDE	1.30	0.44–4.70	1.30	0.57–4.00	0.174	0.09	100
HCB	0.10	0.05–0.19	0.11	0.06–0.18	0.522	0.04	100
*t*-NC	0.04	0.02–0.09	0.04	0.02–0.08	0.020	0.01	100
*β*-HCH	0.12	0.06–0.25	0.13	0.07–0.28	0.007	0.01	99
Lipid-adjusted (ng/g lipids)							
PCB 153	79.9	46.8–146	117	69.4–179	< 0.001	–	–
*p,p’*-DDE	211	78.0–844	244	101–662	0.101	–	–
HCB	17.0	10.7–30.1	18.4	10.3–30.9	0.261	–	–
*t*-NC	6.77	3.62–14.5	6.28	3.25–12.4	0.034	–	–
*β*-HCH	21.2	10.7–39.5	25.0	12.6–44.6	< 0.001	–	–

*PFAS* perfluoroalkyl substance, *OC* organochlorine, *IQR* inter-quartile range, *SD* standard deviation, *LOD* limit of detection, *% > LOD* percentage of serum samples above LOD, *PFOA* perfluorooctanoate, *PFOS* perfluorooctane sulfonate, *PCB* polychlorinated biphenyl, *p,p’-DDE p,p’*-dichlorodiphenyldichloroethane, HCB

^a^*p*-values for comparison between the countries using Mann-Whitney U test

Adjusted linear and logistic associations between maternal serum concentrations of PFASs and OCs, and measures of child adiposity at 5-year follow-up are shown in Table 3. These results are stratified by country of residence based on some indication of effect modification by country (p_interaction_ = 0.039) between maternal serum PFOS concentrations and offspring BMI z-scores as well as child overweight/obesity at 5-year follow-up (p_interaction_ = 0.098). In the total cohort, adjusted BMI-for-age-and-sex z-score increased by 0.18 (95% CI: 0.01–0.35) and adjusted triceps skinfold z-score increased by 0.15 (95% CI: 0.02–0.27) for each ln-unit increase in maternal serum PFOS concentrations. For each ln-unit increase in maternal serum PFOS concentration, the adjusted OR for child overweight/obesity was 2.04 (95% CI: 1.11–3.74). The data also suggests positive associations between maternal serum PFOA concentrations and child BMI z-scores, triceps skinfolds z-scores and child overweight/obesity at 5-year follow-up (Table 3).

**Table 3:** Adjusted associations between ln-units of maternal serum POPs and BMI-for-age-and-sex z-scores, subscapular and triceps skinfold z-scores (*β*s and 95% CIs) and child obesity/overweight (OR and 95% CI) at 5 years of age, overall and by country

Maternal serum POPs	Child outcomes at five-year follow-up (*n* = 412)			
	All participants (*n* = 412)^a^	Norway (*n* = 254)^b^	Sweden (*n* = 158)^b^	
	BMI-for-age-and-sex z-score			
	β (95% CI)			*p*_int_^c^
PFOS	0.18 (0.01, 0.35)	0.30 (0.08, 0.51)	−0.11 (−0.41, 0.19)	0.039
PFOA	0.18 (−0.03, 0.39)	0.32 (0.05, 0.60)	−0.07 (−0.41, 0.27)	0.129
PCB 153	0.30 (−0.03, 0.63)	0.45 (0.03, 0.87)	0.11 (−0.43, 0.65)	0.509
*p,p’*-DDE	0.03 (−0.12, 0.18)	0.04 (−0.15, 0.23)	0.02 (−0.24, 0.29)	0.932
HCB	0.16 (−0.13, 0.45)	0.36 (−0.03, 0.75)	−0.11 (−0.56, 0.34)	0.154
b-HCH	0.17 (−0.08, 0.43)	0.17 (−0.16, 0.51)	0.14 (−0.26, 0.55)	0.823
t-NC	0.11 (−0.13, 0.35)	0.23 (−0.07, 0.54)	−0.12 (−0.53, 0.29)	0.380
	Triceps skinfold z-score			
	β (95% CI)			
PFOS	0.15 (0.02, 0.27)	0.20 (0.06, 0.35)	−0.02 (−0.27, 0.22)	0.255
PFOA	0.14 (−0.02, 0.29)	0.24 (0.05, 0.42)	−0.05 (−0.33, 0.23)	0.223
PCB 153	−0.08 (−0.31, 0.16)	0.02 (−0.27, 0.31)	−0.33 (−0.77, 0.11)	0.638
*p,p’*-DDE	−0.04 (−0.15, 0.07)	0.004 (−0.13, 0.14)	−0.12 (−0.33, 0.10)	0.905
HCB	0.02 (−0.20, 0.24)	0.07 (−0.20, 0.35)	−0.11 (−0.48, 0.26)	0.914
b-HCH	0.10 (−0.08, 0.29)	0.10 (−0.13, 0.32)	0.07 (−0.27, 0.41)	0.271
t-NC	−0.03 (−0.20, 0.15)	0.05 (−0.16, 0.25)	−0.32 (−0.64, 0.02)	0.497
	Subscapular skinfold z-score			
	β (95% CI)			
PFOS	0.07 (−0.07, 0.20)	0.12 (−0.04, 0.29)	−0.07 (−0.35, 0.22)	0.304
PFOA	−0.03 (−0.20, 0.15)	0.04 (−0.18, 0.25)	−0.11 (−0.42, 0.21)	0.470
PCB 153	−0.05 (−0.32, 0.23)	0.07 (−0.26, 0.41)	−0.30 (−0.80, 0.20)	0.432
*p,p’*-DDE	−0.07 (−0.19, 0.06)	−0.04 (−0.18, 0.11)	−0.10 (−0.34, 0.14)	0.917
HCB	−0.08 (−0.33, 0.17)	−0.05 (−0.36, 0.27)	−0.21 (−0.62, 0.20)	0.977
b-HCH	0.14 (−0.07, 0.35)	0.10 (−0.16, 0.36)	0.17 (−0.21, 0.55)	0.528
t-NC	−0.13 (−0.34, 0.07)	−0.08 (−0.32, 0.16)	−0.34 (−0.71, 0.04)	0.698
	Overweight (≥ 85th percentile**)**			
	OR (95% CI)			
N overweight/total	52/412 (13%)	36/254 (14%)	16/158 (10%)	
PFOS	2.04 (1.11–3.74)	2.96 (1.42–6.15)	0.71 (0.21–2.45)	0.098
PFOA	1.61 (0.75–3.46)	2.90 (1.10–7.63)	0.50 (0.12–2.06)	0.119
PCB 153	1.37 (0.42–4.49)	2.13 (0.49–9.26)	0.60 (0.06–6.03)	0.944
*p,p’*-DDE	0.88 (0.52–1.49)	1.11 (0.58–2.14)	0.61 (0.20–1.83)	0.792
HCB	1.51 (0.55–4.13)	2.30 (0.62–8.50)	0.60 (0.10–3.62)	0.806
b-HCH	2.14 (0.86–5.32)	2.17 (0.67–6.99)	1.56 (0.29–8.39)	0.529
t-NC	1.44 (0.63–3.31)	1.69 (0.62–4.62)	0.78 (0.15–1.19)	0.745

^a^Adjusted for maternal age, education, smoking at conception, pre-pregnancy BMI, weight gain at 17 weeks, interpregnancy interval, previous breastfeeding duration and country of residence

^b^Adjusted for maternal age, education, smoking at conception, pre-pregnancy BMI, weight gain at 17 weeks, interpregnancy interval and previous breastfeeding duration

^c^*p*-value for interaction between exposure and country

Among Norwegian children, we observed increased BMI-for-age-and-sex z-scores for each ln-unit increase in maternal serum PFOS concentration (β:0.30 (95% CI: 0.08, 0.51), and each ln-unit increase in maternal serum PFOA concentration (β:0.32 (95% CI: 0.05, 0.60) (Table 3). Norwegian children also showed increased triceps skinfold z-scores per ln-unit increase in maternal serum PFOS concentration (β:0.20, 95% CI: 0.06, 0.35) and maternal serum PFOA concentration (β: 0.24, 95% CI: 0.05, 0.42). BMI z-scores increased by 0.45 (95% CI: 0.03, 0.87) for each ln-unit increase in maternal serum PCB 153 concentration in the Norwegian part. No associations were observed among Swedish participants.

In adjusted logistic regression, we observed no overall association between maternal serum POP concentrations and child adiposity or overweight/obesity in the pooled analyses. Among Norwegian children, we observed increased odds for child overweight/obesity at 5-year follow-up for each ln-unit increase in maternal serum PFOS concentration (OR_adjusted_: 2.96, 95% CI: 1.42–6.15) and maternal serum PFOA concentration (OR_adjusted_: 2.90, 95% CI: 1.10–7.63).

**Fig. 2:**
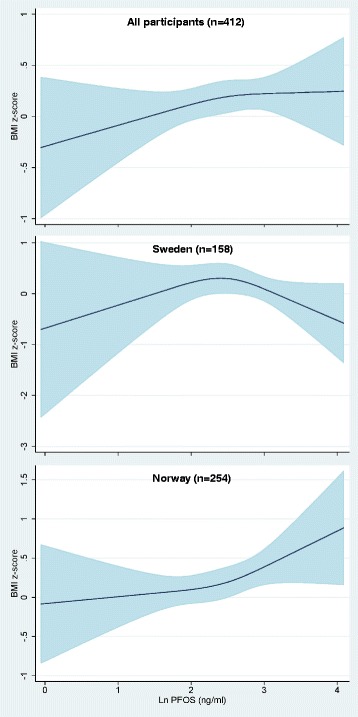
Adjusted restricted cubic spline with 3 knots for maternal serum PFOS concentrations and offspring BMI-for-age-and-sex z-score at 5-year follow-up, overall and stratified by country. All models are adjusted adjusted for maternal age, pre-pregnancy body mass index, education, smoking status during pregnancy, inter-pregnancy interval, previous breastfeeding duration and maternal weight gain up to 17 weeks of gestation. The overall model is additionally adjusted for country of origin. The solid line represents the mean BMI z-score and the shaded area represents the 95% confidence interval

**Fig. 3:**
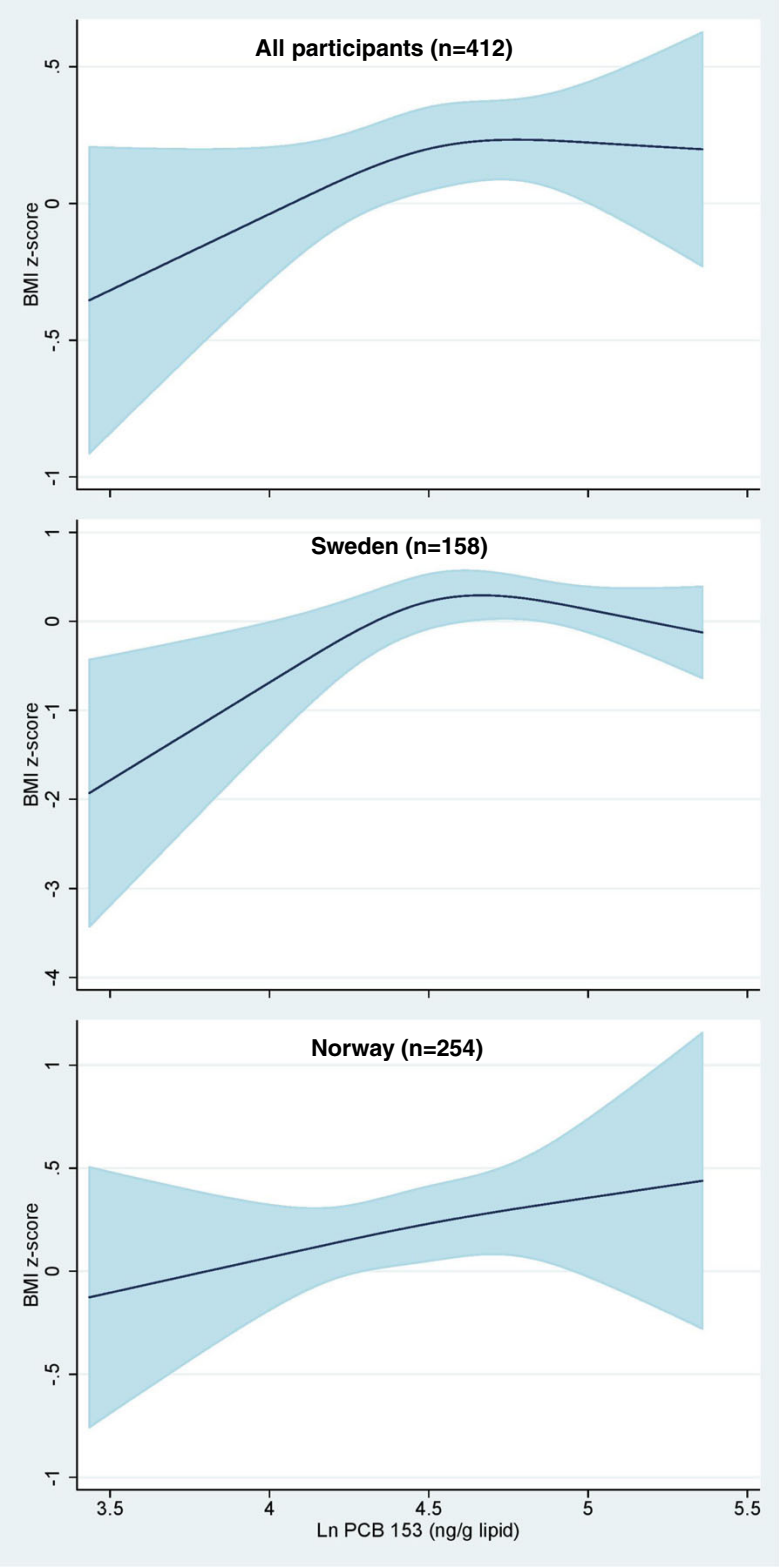
Adjusted restricted cubic spline with 3 knots for maternal serum PCB 153 concentrations and offspring BMI-for-age-and-sex z-score at 5–year follow-up, overall and stratified by country. All models are adjusted for maternal age, pre-pregnancy body mass index, education, smoking status during pregnancy, inter-pregnancy interval, previous breastfeeding duration and maternal weight gain up to 17 weeks of gestation. The overall model are additionally adjusted for country of origin. The solid line represents the mean BMI z-score and the shaded area represents the 95% confidence interval

To examine potential NMDR relationship between maternal serum POP concentrations and child overweight/obesity outcomes, we used a restricted 3-knot cubic spline model. Among Swedish participants, we observed some evidence of a NMDR relationship between maternal serum PFOS concentration and offspring BMI z-scores at 5-year follow-up (*p* = 0.09 for non-linearity, Fig. 2). We found also some indications of a NMDR relationship between maternal serum PCB 153 concentrations and offspring BMI z-scores at 5-year follow-up (*p* = 0.02 for non-linearity, Fig. 3) in the Swedish part of the study.

We tested the generalizability of our results in a stratum-weighted analysis that accounted for the original SGA study design that included a higher proportion of SGA births, a lower prevalence of maternal overweight, and a higher prevalence of smoking mothers at conception, compared to the general pregnant population. Our stratum-weighted analysis did not substantially change our reported results (Additional file 1: Table S1). Adjustment for maternal fish intake among the Norwegian women also did not change the estimates (data not shown). Mutual adjustment between maternal serum PFOS and PCB 153 concentrations, and maternal serum PFOA and PCB 153 concentrations did not change the current estimates. However, adding both maternal serum PFOS and PFOA concentrations into the same model resulted in some attenuation of the estimates probably due to high correlation between the PFASs (Additional file 1: Table S2).

## Discussion

In this prospective cohort study of 412 mother-child pairs from Norway and Sweden, we observed positive associations between maternal serum PFAS concentrations and child BMI and triceps skinfold z-scores, as well as child overweight/obesity at 5-year follow-up, particularly among Norwegian women. We also found evidence of NMDR relationships between maternal serum PFOS and PCB 153 concentrations and offspring BMI z-scores among Swedish participants.

Evidence of prenatal exposure to PFASs and child postnatal growth and obesity is limited and the results have been inconsistent. A Danish study with maternal plasma PFAS concentrations like ours (median PFOS: 10.8 ng/ml, PFOA: 1.3 ng/ml), found positive associations between PFOS and PFOA concentrations and waist-to-height ratio in 5–9-year-old girls and boys [12]. However, studies with higher maternal PFAS concentrations have been more inconsistent. Three studies with higher maternal plasma PFAS concentrations than our study (median PFOS: 19.6–24.8 ng/ml, PFOA: 3.7–5.6 ng/ml) showed positive associations between increasing PFAS concentrations and measures of child obesity (10, 11, 14). However, another Danish study with even higher maternal plasma PFAS concentrations (median PFOS: 33 ng/ml, PFOA: 5.2 ng/ml) found no or inverse associations between increasing PFAS concentrations and BMI or waist circumference among 7-year-old children [16]. Another study with higher maternal serum PFOA concentrations (median: 5.3 ng/ml) than ours, observed a NMDR relationship between increasing PFOA concentrations and BMI at 8 years of age [13]. From this, we may speculate that low maternal serum or plasma PFAS concentrations show positive associations with child obesity, while higher maternal serum or plasma PFAS concentrations show positive, negative, or NMDR relationships with child obesity, depending on the range of PFAS concentrations measured in the study population. This is in agreement with a recent review suggesting that NMRD relationships may be observed with EDCs, and that the effects of high doses EDCs cannot predict the effects of EDCs at lower doses [40]. Possible mechanisms for these NMDR relationships include cytotoxicity, cell- and tissue-specific receptors and cofactors, receptor selectivity, receptor down-regulation and desensitization, receptor competition and endocrine negative feedback loops [40]. This is consistent with our different findings between Norwegian participants (with lower maternal serum PFAS concentrations) compared to Swedish participants (with higher maternal serum PFAS concentrations). These results support a potential cytotoxic effect of high levels of PFASs in utero that can result in growth restricted offspring, which is consistent with the positive associations we found between maternal PFAS concentrations and SGA birth among the Swedish participants in our study [35]. Consequently, this effect may distort the association between maternal serum PFAS concentrations and child obesity at 5-year follow-up. However, a possible obesogenic effect may appear later in the latter development of growth restricted offspring. NMDR relationships have also been proposed for associations between maternal serum PCB concentrations and offspring growth and obesity [41]. A recent review categorized *n* = 9 prospective cohort studies according to the level of maternal serum PCB concentrations whereby the authors suggested that low maternal serum PCB concentration (PCBs < 1000 ng/g lipids) was associated with increased offspring BMI or body weight, while high maternal serum PCB concentration (PCBs > 4000 ng/g lipids) was associated with decreased offspring BMI or body weight [41]. Taken together with our finding of a NMDR relationship between maternal serum PCB 153 concentrations and child overweight/obesity at 5-year follow-up among Swedish only participants, there is some indication that low maternal serum exposure concentrations may lead to offspring obesity, while higher concentrations of PCBs may exert cytotoxic effects on the fetus, resulting in poor fetal growth and development. However, it is difficult to compare concentration ranges in our study with results from this review, as the review considered total maternal serum PCB concentrations [41].

The current study has several strengths including the relatively substantial number of mother-child pairs (*n* = 412). We measured maternal serum PFAS and OC concentrations early in pregnancy, and evaluated mothers and children throughout pregnancy, infancy and into early childhood using detailed clinically based outcome assessments. The use of standardized anthropometric measurements may reduce possible misclassification and enhance the statistical precision of our estimates. To our knowledge, only one previous study has assessed the relationship between maternal serum prenatal PFAS concentrations and offspring triceps and subscapular skinfold thickness [11]. Studies that measure only BMI are limited by the fact that BMI is not a direct measure of fat distribution. As such, children with the same BMI may differ considerably in total amount of body fat [42]. Skinfold thickness, as applied in our study, is used as a measure of subcutaneous fat, which has been reported to be highly correlated with total amount body fat [19, 43]. We were also able to examine and/or adjust for several important prenatal and postnatal factors. Our study is one of few studies to investigate a variety of maternal serum PFAS and OC exposures.

The current study also has some limitations. Although we included a range of covariates in multivariate models, we cannot rule out possible residual confounding related to socio-economic and lifestyle differences between high-consumers of seafood in Norway compared to Sweden. However, adjustment for maternal fish intake among Norwegian participants did not change the results. The contamination pattern and POP concentrations in seafood from the Baltic Sea on the East Coast of Sweden is different compared to the seafood consumed in Norway [22]. Thus, the possible nutrient/contaminant interaction might be different in the two countries. We acknowledge that SGA births were overrepresented in our study cohort compared to the general pregnant population, which may introduce selection bias or problems with generalizability. Also, the high prevalence of maternal smoking and the low prevalence of maternal overweight in our study compared to recent pregnant populations [44], might distort the estimates. However, results from stratum-weighted analyses showed no change in reported results. Still, results from this study may not be generalizable to primiparous women, as only parous women were eligible for study inclusion. Although skinfold measurements in children are more correlated with body fat mass, they are prone to intra- and inter-observer errors [32]. We had no information about the inter-observer reliability, but we assume that measurement precision was un-correlated with maternal serum EDC concentrations. Persistent and bio-accumulative chemicals with comparable properties are highly correlated. Therefore, our point estimates may be subject to residual confounding due to some unmeasured chemicals (e.g. lead) in our analyses. Finally, we must interpret our country-specific results with caution due to small numbers.

## Conclusion

Our study shows that increasing maternal serum PFAS concentrations were associated with increasing child BMI and triceps skinfold z-scores, in addition to child overweight/obesity at 5-year follow-up, but this association may differ geographically and by maternal serum PFAS concentration. Our results also highlight the importance of assessing NMDR relationships for POP exposures. More prospective studies on the association between maternal serum POP concentrations and overweight/obesity among older children and adults are needed.

## Additional file

Additional file 1: Supplementary figures, tables and information. (DOCX 323 kb)

## Abbreviations

AMAP: Arctic Monitoring and Assessment Programme
BMI: body mass index
CI: confidence interval
DAG: directed acyclic graph
EDC: endocrine disrupting chemical
HCB: hexachlorobenzene
LOD: limit of detection
NICHD: National Institute of Child Health and Human Development
NILU: Norwegian Institute for Air Research
NMDR: non-monotonic dose-response
OC: organochlorine
OR: odds ratio
*p,p’*-DDE: *p,p’*-dichlorodiphenyldichloroethane
PCB: polychlorinated biphenyl
PFAS: perfluoroalkyl substance
PFOA: perfluorooctanoate
PFOS: perfluorooctane sulfonate
POP: persistent organic pollutant
SGA: small for gestational age
*t*-NC: trans-nonachlor
UHPLC-MS/MS: ultra-high pressure liquid chromatography triple-quadrupole mass-spectometry
WHO: World Health Organization.
*β-*HCH: beta-hexachlorohexane
