# Supplementary material for: Prenatal exposure to persistent organic pollutants and child overweight/obesity at 5-year follow-up: a prospective cohort study
Source: Environ Health. 2018 Jan 18;17:9. doi: 10.1186/s12940-017-0338-x (PMC5774128; doi:10.1186/s12940-017-0338-x)
Supplement: Additional file 1: — Supplementary figures, tables and information. (DOCX 323 kb) [file 12940_2017_338_MOESM1_ESM.docx]

**Prenatal exposure to persistent organic pollutants and child overweight/obesity at 5-year follow-up: a prospective cohort study**

Hilde B. Lauritzen, Tricia L. Larose, Torbjørn Øien, Torkjel M. Sandanger, Jon Ø. Odland, Margot van de Bor and Geir W. Jacobsen

**Supplementary Figure S1**. Directed acyclic graph (DAG) of the relationships between maternal serum POP levels and postnatal growth and obesity.

**Supplementary description S1.** Methods for weighting variables in sensitivity analyses.

**Supplementary description S2**. Methods for assessing fish consumption at gestational week 17-20.

**Supplementary Table S1.** Adjusted associations between ln-units of PFASs and BMI-for-age-and-sex z-scores, triceps skinfold z-scores (βs and 95% CIs) and overweight (OR and 95% CI) at 5 years of age, *un-weighted and weighted* according to prevalence of SGA offspring (10%), maternal overweight (30%) and smoking during pregnancy (3%), including all participants (n=412) and the Norwegian part (n=254).

**Supplementary Table S2.** Adjusted associations between ln-units of POPs and BMI-for-age-and-sex z-scores (β and 95% CI) and overweight (OR and 95% CI) at 5 years of age, with mutual adjustment for PFOS, PFOA and PCB 153, all participants (n=412) and Norwegian part (n=254).

**
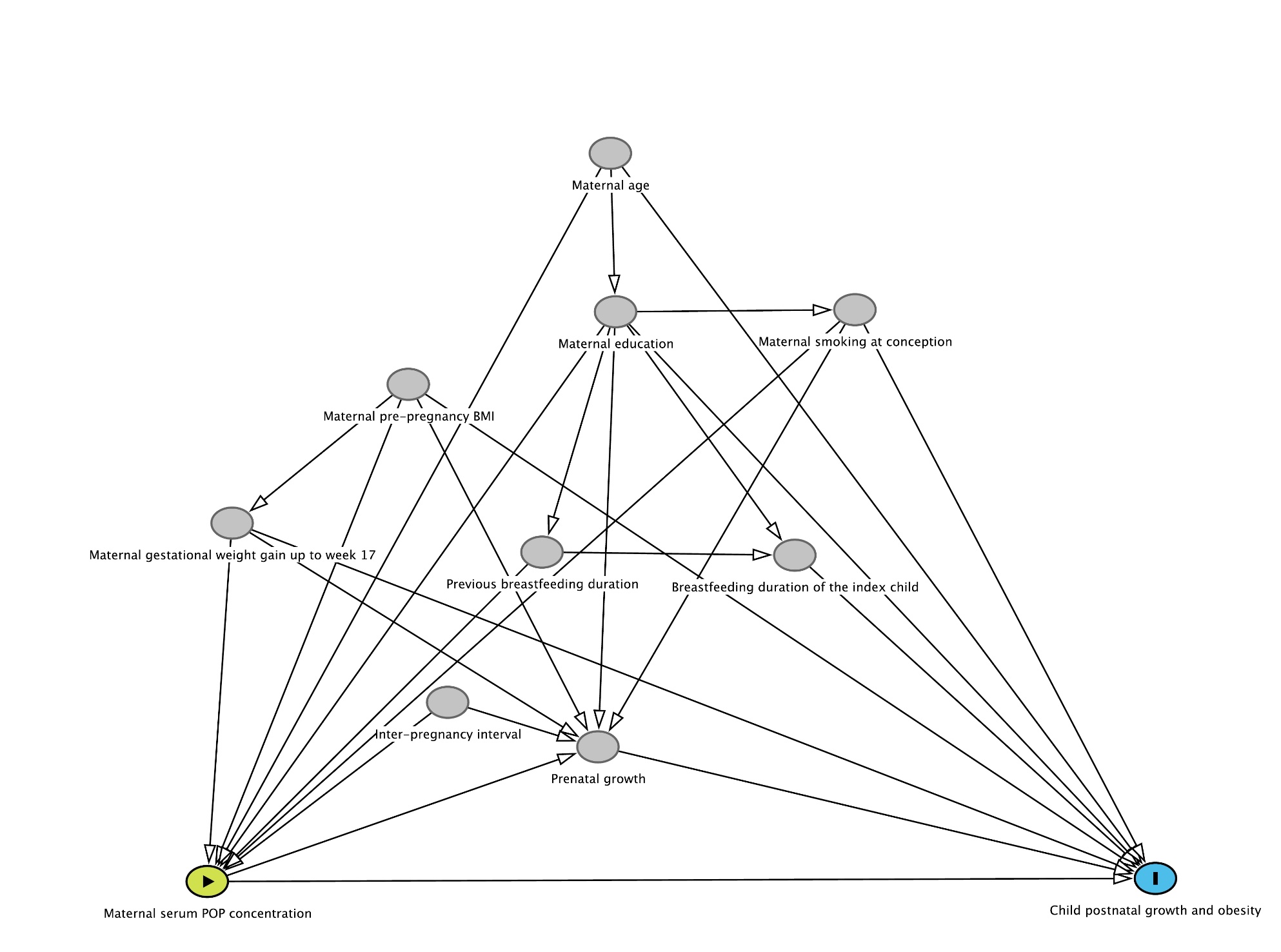
Supplementary Figure S1. Directed acyclic graph (DAG) of the relationships between maternal serum POP levels and postnatal growth and obesity**

**Supplementary description S1. Methods for weighting variables in sensitivity analyses**

Our cohort consisted of 33% SGA children in contrast to 10% in a general population, and to ensure generalizability, we did stratum-weighted sensitivity analyses where weights were the inverse probability of selection [1]. This means that the under-represented group get a weight larger than 1, and those in the over-represented group get a weight smaller than 1. We computed the weights by this formula: *percentage in general population divided by percentage in study sample*. Hence, SGA children were weighted 0.30 (=10% divided by 33%) and non-SGA children were weighted 1.35 (=90% divided by 67%) in these analyses.

We also performed weighted sensitivity analyses based on smoking prevalence and maternal overweight prevalence, which differed in our cohort compared to the general pregnant population today. In the former analyses, we weighted smokers 0.27 (=12% divided by 45%) and non-smokers 1.60 (=88% divided by 55%), and in the latter, we weighted mothers with overweight 3.26 (=31% divided by 9.5%), normal weight 0.83 (=66% divided by 80%) and underweight 0.29 (=3% divided by 10.5%). Prevalence of smoking during pregnancy and pre-pregnancy overweight was based on a prospective population-based pregnancy cohort study conducted in Norway from 1999-2008 [2].

**Supplementary description S2. Methods for assessing fish consumption at gestational week 17-20.**

At gestational week 17-20, three days of dietary records were collected among the Norwegian women [3]. Data were collected during the same three weekdays (Tuesday, Wednesday and Thursday). The amounts of food consumed were given in household measures, supplemented by food models presented as booklet with full scale drawings. Internal validity was tested against a food frequency questionnaire in a comparable group of non-pregnant Norwegian women. Maternal fish consumption was calculated as gram consumed of lean and fatty fish, shellfish and fish spread, and categorized as 0, 1-50 and >50 grams per day.

| **Supplementary Table S1. Adjusted associations between ln-units of PFASs and BMI-for-age-and-sex z-scores, triceps skinfold z-scores (βs and 95% CIs) and overweight (OR and 95% CI) at 5 years of age, *un-weighted and weighted* according to prevalence of SGA offspring (10%), maternal overweight (30%) and smoking during pregnancy (12%), including all participants (n=412) and the Norwegian part (n=254)** | | | | | | |
| --- | --- | --- | --- | --- | --- | --- |
| **Maternal serum levels** | **All participants (n=412)** | | | **Norway (n=254)** | | |
|  | **BMI z-score^1^** | **Triceps skinfold z-score^1^** | **Overweight (≥85th percentile)^1^** | **BMI z-score^2^** | **Triceps skinfold z-score^2^** | **Overweight (≥85th percentile)^2^** |
|  | β (95% CI) | β (95% CI) | OR (95% CI) | β (95% CI) | β (95% CI) | OR (95% CI) |
| **PFOS** |  |  |  |  |  |  |
| A | **0.18 (0.01, 0.35)** | **0.15 (0.02, 0.27)** | **2.04 (1.11-3.74)** | **0.30 (0.08, 0.51)** | **0.20 (0.06, 0.35)** | **2.96 (1.42-6.15)** |
| B | **0.19 (0.02, 0.35)** | **0.19 (0.07, 0.31)** | **2.04 (1.13-3.67)** | **0.29 (0.08, 0.49)** | **0.25 (0.11, 0.39)** | **3.00 (1.47-6.13)** |
| C | **0.27 (0.10, 0.45)** | **0.19 (0.06, 0.32)** | **1.98 (1.12-3.51)** | **0.43 (0.20, 0.65)** | **0.25 (0.10, 0.41)** | **2.95 (1.48-5.87)** |
| D | 0.15 (-0.01, 0.32) | **0.20 (0.07, 0.32)** | **3.05 (1.49-6.25)** | **0.24 (0.02, 0.46)** | **0.20 (0.04, 0.35)** | **6.87 (2.50-18.9)** |
| **PFOA** |  |  |  |  |  |  |
| A | 0.18 (-0.03, 0.39) | 0.14 (-0.02, 0.29) | 1.61 (0.75-3.46) | **0.32 (0.05, 0.60)** | **0.24 (0.05, 0.42)** | **2.90 (1.10-7.63)** |
| B | 0.14 (-0.08, 0.35) | **0.18 (0.02, 0.33)** | 1.40 (0.66-3.00) | 0.21 (-0.06, 0.48) | **0.27 (0.08, 0.46)** | 2.32 (0.90-5.95) |
| C | **0.22 (0.001, 0.44)** | **0.19 (0.02, 0.35)** | 1.81 (0.84-3.89) | **0.33 (0.03, 0.63)** | **0.28 (0.08, 0.48)** | **3.03 (1.14-8.11)** |
| D | 0.16 (-0.05, 0.37) | **0.19 (0.03, 0.35)** | 2.10 (0.91-4.86) | **0.29 (0.01, 0.57)** | **0.24 (0.04, 0.44)** | **4.52 (1.42-14.3)** |
| ^1^Adjusted for maternal age, education, smoking at conception, pre-pregnancy BMI, maternal weight gain at 17 weeks of gestation, interpregnancy interval, previous breastfeeding duration and country of residence | | | | | | |
| ^2^Adjusted for maternal age, education, smoking at conception, pre-pregnancy BMI, maternal weight gain at 17 weeks of gestation, interpregnancy interval and previous breastfeeding duration | | | | | | |
| A: Un-weighted analysis | |  |  |  |  |  |
| B: Weighted analysis; SGA offspring = 10% | | |  |  |  |  |
| C: Weighted analysis; maternal overweight = 30% | | |  |  |  |  |
| D: Weighted analysis; smoking during pregnancy = 12% | | | |  |  |  |

| **Supplementary Table S2. Adjusted associations between ln-units of POPs and BMI-for-age-and-sex z-scores (β and 95% CI) and overweight (OR and 95% CI) at 5 years of age, with mutual adjustment for PFOS, PFOA and PCB 153, all participants (n=412) and Norwegian part (n=254)** | | |
| --- | --- | --- |
| **Maternal serum POPs** |  |  |
|  | **All participants (n=412)^1^** | **Norway (n=254)^2^** |
|  | **BMI-for-age-and-sex z-score** | |
|  | β (95% CI) | |
| **PFOS** |  |  |
| Adjusted | **0.18 (0.01, 0.35)** | **0.30 (0.08, 0.51)** |
| +PCB 153 | 0.17 (-0.01, 0.34) | **0.28 (0.07, 0.49)** |
| +PCB 153 and PFOA | 0.12 (-0.11, 0.35) | 0.22 (-0.07, 0.50) |
| **PFOA** |  |  |
| Adjusted | 0.18 (-0.03, 0.39) | **0.32 (0.05, 0.60)** |
| +PCB 153 | 0.16 (-0.05, 0.18) | **0.29 (0.02, 0.57)** |
| +PCB 153 and PFOS | 0.06 (-0.23, 0.35) | 0.10 (-0.27, 0.47) |
| **PCB 153** |  |  |
| Adjusted | 0.30 (-0.03, 0.63) | **0.45 (0.03, 0.87)** |
| +PFOS | 0.30 (-0.03, 0.63) | *0.41 (-0.01, 0.83)* |
| +PFOS and PFOA | 0.29 (-0.04, 0.62) | *0.42 (-0.002, 0.83)* |
|  | **Overweight (≥85th percentile)** | |
|  | β (95% CI) | |
| **PFOS** |  |  |
| Adjusted | **2.04 (1.11-3.74)** | **2.96 (1.42-6.15)** |
| +PCB 153 | 1.72 (0.97-3.06) | **2.90 (1.39-6.08)** |
| +PCB 153 and PFOA | 1.97 (0.88-4.44) | 2.64 (0.97-7.20) |
| **PFOA** |  |  |
| Adjusted | 1.61 (0.75-3.46) | **2.90 (1.10-7.63)** |
| +PCB 153 | 1.44 (0.68-3.05) | **2.77 (1.04-7.39)** |
| +PCB 153 and PFOS | 0.79 (0.28-2.23) | 1.21 (0.33-4.47) |
| **PCB 153** |  |  |
| Adjusted | 1.37 (0.42-4.49) | 2.13 (0.49-9.26) |
| +PFOS | 0.67 (0.25-1.83) | 1.75 (0.38-8.07) |
| +PFOS and PFOA | 0.78 (0.27-2.23) | 1.75 (0.34-8.99) |
| ^1^Adjusted for maternal age, education, smoking at conception, pre-pregnancy BMI, weight gain at 17 weeks, inter-pregnancy interval, previous breastfeeding duration and country of residence. | | |
| ^2^Adjusted for maternal age, education, smoking at conception, pre-pregnancy BMI, weight gain at 17 weeks, inter-pregnancy interval and previous breastfeeding duration. | | |

**References**

1. Richardson DB, Rzehak P, Klenk J, Weiland SK. Analyses of case-control data for additional outcomes. Epidemiology. 2007; doi:10.1097/EDE.0b013e318060d25c

2. Sorbye LM, Klungsoyr K, Samdal O, Owe KM, Morken NH. Pre-pregnant body mass index and recreational physical activity: effects on perinatal mortality in a prospective pregnancy cohort. Bjog. 2015; doi:10.1111/1471-0528.13290

3. Buzzard M. 24-hour dietary recall and food record methods. MONOGRAPHS IN EPIDEMIOLOGY AND BIOSTATISTICS. 1998:50-73.
